# Correction: Bulatov et al. Camelpox Virus in Western Kazakhstan: Assessment of the Role of Local Fauna as Reservoirs of Infection. *Viruses* 2024, *16*, 1626

**DOI:** 10.3390/v18060689

**Published:** 2026-06-22

**Authors:** Yerbol Bulatov, Sholpan Turyskeldy, Ruslan Abitayev, Abdurakhman Usembai, Zhanna Sametova, Zhanat Kondybayeva, Alina Kurmasheva, Dana Mazbayeva, Asselya Kyrgyzbayeva, Kamshat Shorayeva, Zhanat Amanova, Dariya Toktyrova

**Affiliations:** 1 Research Institute for Biological Safety Problems, Gvardeiskiy 080409, Kazakhstan; ye.bulatov@biosafety.kz (Y.B.); sh.smankizi@biosafety.kz (S.T.); r.abitaev@biosafety.kz (R.A.); a.ussenbay@biosafety.kz (A.U.); zh.sametova@biosafety.kz (Z.S.); zh.kondybaeva@biosafety.kz (Z.K.); a.kurmasheva@biosafety.kz (A.K.); mazbayevad@mail.ru (D.M.); asselya91@icloud.com (A.K.); k.shorayeva@biosafety.kz (K.S.); zh.amanova@biosafety.kz (Z.A.); 2 Faculty of Biology and Biotechnology, Department of Biotechnology, Al-Farabi Kazakh National University, Almaty 050040, Kazakhstan

## Additional Affiliation

In the published publication [1], there was an error regarding the affiliation for Sholpan Turyskeldy. In addition to affiliation 1, the updated affiliations should include: Faculty of Biology and Biotechnology, Department of Biotechnology, Al-Farabi Kazakh National University, Almaty 050040, Kazakhstan. The authors state that the scientific conclusions are unaffected. This correction was approved by the Academic Editor. The original publication has also been updated.
